# The effect of postpartum nursing guidance on early pelvic floor dysfunction recovery in women of advanced maternal age: a randomized controlled trial

**DOI:** 10.3389/fmed.2024.1397258

**Published:** 2024-07-31

**Authors:** Qingyu Huang, Junfang Tang, Dingyuan Zeng, Yu Zhang, Ting Ying

**Affiliations:** ^1^Department of Women’s Rehabilitation, Liuzhou Maternity and Child Healthcare Hospital, Liuzhou, Guangxi, China; ^2^Key Laboratory of Birth Cohort Study for Aged Pregnant Women of Guangxi Health Commission, Liuzhou, Guangxi, China

**Keywords:** advanced maternal age, postpartum nursing guidance, pelvic floor function, rehabilitation, pelvic floor rehabilitation

## Abstract

**Objective:**

This study aimed to investigate the efficacy of postpartum nursing guidance in the treatment of early pelvic floor dysfunction (PFD) in women of advanced maternal age.

**Methods:**

A total of 146 patients of advanced maternal age admitted to our hospital between January and December 2021 were enrolled in this study and randomly divided into two groups: the control group and the experimental group, with 73 patients in each group. Parturients in the control group received routine pelvic floor rehabilitation treatment, whereas those in the experimental group were given individualized postpartum nursing guidance alongside routine pelvic floor rehabilitation treatment. The recovery of pelvic floor muscle (PFM) strength, the incidence of PFD diseases and nursing satisfaction were compared between the two groups after 3 months of treatment.

**Results:**

The enhancement of PFM strength in the experimental group significantly surpassed that in the control group. Furthermore, the experimental group exhibited a notably lower overall occurrence of PFD and significantly greater maternal satisfaction compared with the control group, and the difference was statistically significant (*p* < 0.05).

**Conclusion:**

Combining postpartum nursing guidance with pelvic floor rehabilitation for women of advanced maternal age represents a treatment regimen deserving of clinical endorsement, as it offers numerous advantages, including substantial improvement in PFM strength, decreased incidence of PFD and enhanced patient satisfaction.

## 1 Introduction

Female pelvic floor dysfunction (PFD) is a group of gynecological diseases with high incidence due to defects, degeneration, injury and dysfunction of the pelvic support structure (1). Data from epidemiological studies reveal high-risk factors for PFD, including age, obesity, constipation, reproductive system inflammation, heredity, pregnancy and delivery, diabetes, connective tissue diseases and neuropathy (2, 3). In the process of vaginal delivery, excessive stretching of maternal pelvic floor muscles (PFMs) and fascia caused by vaginal dilation and injury leads to decreased elasticity of the maternal PFMs and is accompanied by the disruption of muscle fibers and other symptoms (4, 5). Consequently, PFD can severely affect women’s quality of life and mental health (6) and can easily induce other gynaecological diseases (7).

The recent enforcement of China’s universal two-child policy has resulted in a rise in the prevalence of clinically advanced maternal-age cases. With older age and diminished reproductive organ function, women of advanced maternal age exhibit notably higher rates of pregnancy and delivery complications, as well as pelvic floor injuries, compared with non-advanced maternal-age cases (8). Research indicates that advancing age is a contributing factor to the onset and progression of PFD (9). Therefore, it is important to conduct studies on rehabilitation treatment for postpartum PFD in women of advanced maternal age. Currently, no surgical treatment for PFD has been proposed in clinical practice. Most patients are advised to undergo rehabilitation training to improve their PFM strength, thus reducing the clinical manifestations of PFD. Furthermore, electrical stimulation combined with biofeedback therapy can restore the pelvic floor function of postpartum women (10, 11).

The female pelvic floor system, including fascia, multi-layer muscles, ligaments and nerves, is crucial for maintaining the normal position of pelvic organs (12, 13). During pregnancy, the enlarged and descended uterus increases the burden on the pelvic floor support system, and failure to restore it promptly after delivery can lead to corresponding dysfunction (14).

Zhou et al. (15) found that age, delivery times, menopause and urinary tract infections are risk factors for PFD, especially during pregnancy and childbirth. Women of advanced maternal age have three superimposed high-risk factors (age, pregnancy, and delivery), making them prone to postpartum PFD, which seriously affects their quality of life. Studies have shown that active pelvic floor rehabilitation, including PFM exercises, biofeedback and electrical stimulation, can effectively improve excessive PFM strength. Training the pelvic floor support structure using the levator ani muscle can restore and strengthen pelvic floor function and promote PFM strength recovery. Furthermore, it is low risk and usually involves no complications (16). It plays a vital role in preventing and treating pelvic floor disorders (17, 18).

However, some studies have highlighted poor compliance with various clinically developed rehabilitation measures among some women due to insufficient knowledge of PFD or significant postpartum mood fluctuations. These studies also suggest that integrating postpartum nursing guidance into routine pelvic floor rehabilitation training, including health education, psychological counseling, Kegel exercises and regular follow-ups, can significantly enhance the coordination of maternal rehabilitation efforts. This approach holds positive significance in ensuring effective rehabilitation outcomes for patients. The efficacy of PFM exercises is closely related to their duration, requiring continuous exercise for more than 3 months. Better results can only be achieved through intensive training and follow-ups (19). Long-term postpartum PFM exercise can significantly improve PFM strength, which is of great importance for improving PFD symptoms and preventing urinary incontinence (20). One study (21) highlighted pessimistic postpartum PFM training (PFMT) adherence and certain limitations in addressing personalized nursing needs during the puerperium. Several studies have emphasized the crucial role of providing comprehensive and systematic rehabilitation intervention guidance for postpartum women in mitigating disease onset. These efforts have profound significance for national medical resource allocation and long-term reduction of the country’s medical burden (22–26).

A recent comprehensive review by DeLancey et al. (27) highlight vaginal birth as the largest modifiable risk factor for prolapse and stress incontinence, and discusses the multifactorial nature of risk factors for levator injury. The authors suggest potential steps to reduce injuries and emphasize the importance of prenatal education, informed decision-making during labor, and postpartum care and rehabilitation.

We hypothesize that postpartum nursing guidance is an independent predictor of parturient recovery beyond physical therapy alone. This study aims to investigate the effect of postpartum nursing guidance on early pelvic floor function rehabilitation in advanced maternal age to promote the improvement of postpartum pelvic floor function and reduce the occurrence of PFD.

## 2 Materials and methods

### 2.1 General data

This prospective study enrolled pregnant women of advanced maternal age who provided signed written consent. The inclusion criteria were based on the diagnosis of PFD after delivery, which was confirmed by the International Continence Society (ICS) criteria (28). The exclusion criteria were as follows: (1) parturients with a history of pelvic disease surgery; (2) parturients who experienced multiple pregnancies or underwent cesarean section surgeries; (3) parturients with a history of other chronic diseases known to impact pelvic floor function, such as diabetes, hypertension or neurological disorders; and (4) parturients who expressed unwillingness or inability to adhere to the intervention protocol.

A total of 146 parturients of advanced maternal age (aged ≥35 years at delivery) admitted to our hospital between January and December 2021 were included in this study. We included the participants within 1 week after vaginal delivery, as we wanted to assess the early PFD and intervene as soon as possible. The advanced maternal age cohort was randomly divided into two groups using the random number table method: the control group and the experimental group, each comprising 73 parturients.

In the control group, the parturients’ age ranged from 35 to 42 years, with an average of 37.55 ± 1.73 years and a median of 37 years. Gestational age varied from 242 to 286 days, with an average of 268.38 ± 9.43 days. The PFM strength scale indicated grade 0 in 5 cases, grade I in 32 cases, grade II in 26 cases and grade III in 10 cases. Disease types included urinary incontinence in 26 cases and pelvic organ prolapse in 54 cases. For the experimental group, the parturients’ age ranged from 35 to 47 years, with an average of 38.12 ± 2.02 years and a median of 38 years. Gestational age ranged from 239 to 287 days, with an average of 266.79 ± 10.86 days. The PFM strength scale showed grade 0 in 6 cases, grade I in 30 cases, grade II in 26 cases and grade III in 9 cases. Disease types included urinary incontinence in 26 cases and pelvic organ prolapse in 58 cases. There were no statistically significant differences observed between the two groups of parturients regarding general data (*p* > 0.05), which were comparable.

**Figure 1:**
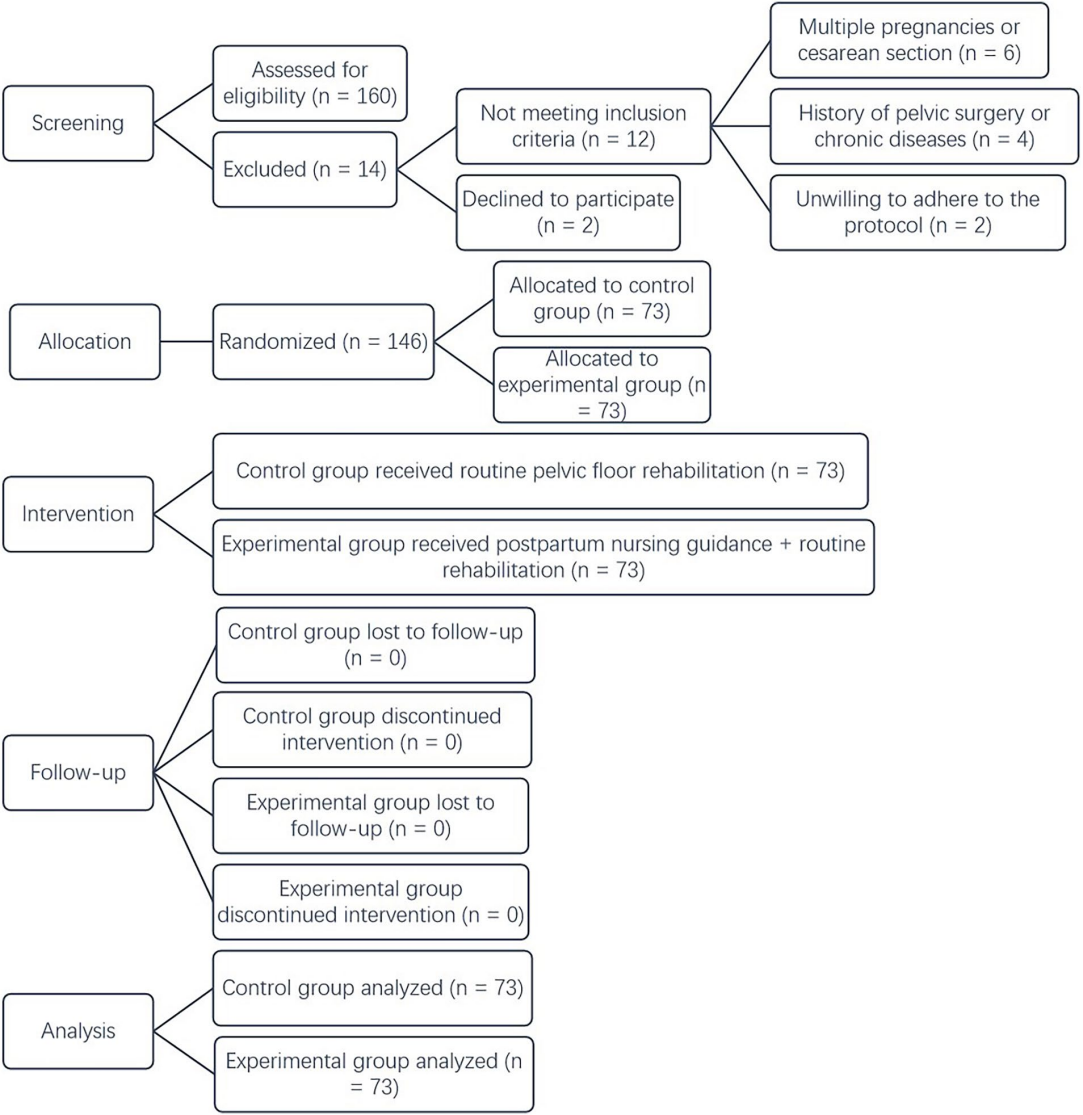
The CONSORT flow diagram.

The median values for age and gestational data for both the control and experimental groups are presented in Table 1. No significant differences were found between the two groups in terms of parity, fetal weight, delivery mode, episiotomy, perineal tear or pelvic floor injury (*p* > 0.05), indicating comparability and balance across these aspects, as depicted in Table 1. The normality of the data in Table 1 was confirmed using the Kolmogorov–Smirnov test, with the results indicating a normal distribution (*p* > 0.05). The study was approved by the ethics committee of our hospital (Approval number: Fast Review-Research-2018-093). The CONSORT flow diagram illustrating the enrollment, allocation, follow-up, and analysis of the study participants is depicted in Figure 1.

**Table 1:** General information of the parturients in both groups.

Characteristic	Control group (*n* = 73)	Experimental group (*n* = 73)	*t-*value	*p-*value
Age (years)	37.55 ± 1.73	38.12 ± 2.02	−2.063	0.067
Gestational age (days)	268.38 ± 9.43	266.79 ± 10.86	0.944	0.347
Parity	1.21 ± 0.42	1.18 ± 0.39	0.517	0.606
Fetal weight (kg)	3.25 ± 0.54	3.21 ± 0.51	0.649	0.517
Delivery mode (Vaginal)	49 (67.12%)	50 (68.49%)	0.474	0.879
Episiotomy (Yes)	32 (43.84%)	30 (41.10%)	0.517	0.606
Perineal tear (Yes)	26 (35.61%)	25 (34.25%)	0.474	0.491
Pelvic floor injury (Yes)	29 (39.73%)	26 (35.61%)	0.153	0.674

### 2.2 Methods

#### 2.2.1 Control group therapy

The parturients in the control group received routine pelvic floor rehabilitation therapy, including low-frequency electrical stimulation and biofeedback therapy, as well as routine postpartum rehabilitation guidance. The specific methods used were as follows: (1) The American neuromuscular electrical stimulation device, Automuse 930 (a therapeutic instrument for electrical muscle stimulation manufactured by Automuse Systems Inc., Salt Lake City, Utah, United States), was selected to deliver different intensities of low-frequency electrical impulses and an individualized biofeedback program. This stimulated the weakened pelvic floor muscles to contract and train rhythmically, strengthened the pelvic floor muscles and provided real-time feedback to effectively promote rehabilitation. Each treatment lasted 30 min and was provided twice a week for a total of 15 treatments. (2) During routine postpartum rehabilitation instruction, parturients were educated on the significance of postpartum pelvic floor rehabilitation to enhance their self-care awareness and self-management skills. A full-time nurse provided health education to each parturient, and a personalized rehabilitation plan was devised based on individual differences to facilitate systematic exercise.

#### 2.2.2 Experimental group therapy

The parturients in the experimental group received the same pelvic floor rehabilitation therapy as the control group, including low-frequency electrical stimulation and biofeedback therapy. In addition to this routine therapy, the experimental group received a postpartum nursing guidance intervention encompassing the following measures: (1) Health education: pelvic floor rehabilitation-related knowledge and cooperation techniques were presented to parturients through visual aids, such as images, videos and health brochures. Additionally, one-on-one consultation services were extended through telephone, WeChat or other means. Prompt responses to parturients’ inquiries and targeted guidance were provided to enhance awareness and compliance with rehabilitation training. (2) Psychological counseling: due to their age and concerns about treatment outcomes, disease recurrence and the demands of childcare, parturients of advanced maternal age are susceptible to anxiety and other negative emotions. To address this, individualized psychological counseling was provided to the parturients based on their educational background and personality traits. This aimed to assist them in accurately recognizing their current pelvic floor issues and understanding the potential outcomes. Additionally, successful cases of pelvic floor rehabilitation were shared with them, enhancing the trust of postpartum women undergoing rehabilitation training and motivating active participation in the exercises. (3) Pelvic floor muscle training, commonly known as Kegel training, involves engaging the anal muscles. Parturients were instructed to contract their PFMs upon exhaling and to relax them while inhaling. Advanced stages of the training included holding the PFM contraction while maintaining normal breathing. They were encouraged to perform focused PFMT without distraction for at least 15 min daily, integrating PFM contractions into their daily activities as a complementary strategy. Additionally, parturients were advised to empty their bladders before starting the exercise by lying flat with the knees naturally bent and open at the width of the iliac crest, followed by a gentle upward tightening of the vagina and anus. The above movements were repeated for 5 min each time at the start of the exercise and gradually increased to 15–25 min, 2–3 times/day. (4) Regular follow-ups: during the treatment, the parturients were supervised and instructed daily to adhere to the PFMT through WeChat. The parturients were scheduled for weekly follow-up sessions to foster positive communication with them and their families, who were urged to provide full support, helping the parturients maintain a positive attitude toward their rehabilitation training.

Three months after completing treatment, the parturients returned to the hospital for follow-up appointments. On average, the experimental group spent 30 min per week with the nurse or engaging in interactive chats.

The differences in treatments received by the control and experimental groups are summarized in Table 2.

**Table 2:** Differences in treatments received by the control and experimental groups.

Treatment	Control group (routine pelvic floor rehabilitation)	Experimental group (routine rehabilitation + postpartum nursing guidance)
e-stim and biofeedback	X	X
Active PFMT	X	X
Health education	X	X
Personalized rehab plan	X	X
One-on-one remote nursing consultation		X
Psychological counseling		X
Successful case sharing		X
Detailed home PFMT instruction		X
Integrating PFM contractions into daily activities		X
Daily communication/instruction via WeChat		X
Weekly follow-up with patient and family		X

### 2.3 Observation indicators

#### 2.3.1 Improvement of pelvic floor muscle strength

Electrophysiological testing of PFM endurance was performed before and 3 months after treatment, and muscle endurance was measured by the percentage of parturients who were able to hold the contraction for 10 s or more, following the recommendations of the ICS report on the terminology of PFM assessment (29). The PFMs were classified into two types according to their function and innervation: Type I muscle fibers, responsible for the maintenance of pelvic organ support and innervated by the pudendal nerve, and Type II muscle fibers, responsible for the rapid contraction and relaxation of the PFM and innervated by the sacral nerve. The muscle endurance of each type was calculated according to the percentage of parturients who met the criterion.

The presence of a levator ani tear, defined as a detachment of the pubovisceral portion of the levator ani muscle from the pubic bone, was assessed by palpating bilaterally during the PFM strength assessment using the pubovisceral muscle palpation technique described by Dietz and Shek (30). The PFM strength was graded using the modified Oxford scale, which ranges from 0 (no contraction) to 5 (strong contraction) (30, 31).

#### 2.3.2 Improvement of urinary incontinence

Improvement in urinary incontinence was assessed and compared between the two groups before treatment and 3 months after treatment. Occasional leakage of urine during laughing, coughing or sneezing was considered mild; frequent leakage of urine during walking, climbing stairs or sexual intercourse was considered moderate; involuntary leakage of urine at rest or while standing was considered severe. Urinary incontinence was evaluated using a questionnaire that was developed based on the International Consultation on Incontinence Questionnaire-Short Form (ICIQ-SF) questionnaire. The questionnaire asked the parturients to rate the frequency and severity of their urinary leakage during different activities, such as laughing, coughing, sneezing, walking, climbing stairs or sexual intercourse. The questionnaire also asked the parturients to rate the impact of their urinary incontinence on their quality of life. It had a total score of 21, with higher scores indicating worse urinary incontinence, which was classified as mild, moderate or severe according to score ranges of 1–5, 6–12, and 13–21, respectively.

#### 2.3.3 Improvement of pelvic organ prolapse

Pelvic organ prolapse was evaluated before treatment and 3 months after treatment. The pelvic organ prolapse quantitative quantification (POP-Q) scale was used to evaluate pelvic organ prolapse in a quiet environment after the participants were instructed to voluntarily empty their bladder, maintain the lithotomy position and sterilize the vulva. Pelvic organ prolapse was graded according to the POP-Q scale as follows: scale 0 indicated no prolapse; scale I indicated that the farthest end of prolapse was located at <−1 cm; scale II indicated that the farthest end of prolapse was located at −1 to +1 cm; scale III indicated that the farthest end of prolapse was located at +1 cm – (total vaginal length – 2) cm; scale IV indicated that the farthest end of prolapse was located at >(total length of vagina – 2) cm.

#### 2.3.4 Nursing satisfaction questionnaire

The nursing satisfaction was measured using a questionnaire that included four rating indices: health education, psychological care, service attitude and rehabilitation effect. Each index was evaluated in three grades: satisfied, basically satisfied and unsatisfied. The questionnaire had a total score of 12, with higher scores indicating higher nursing satisfaction. Satisfaction = (number of satisfied cases + number of basically satisfied cases)/total number of cases × 100%.

### 2.4 Statistical processing

The sample size was determined by a power analysis using G*Power software (v.3.1.9.7). This study assumed a medium effect size of 0.5 for the main outcome of PFM strength. The power was set at 0.8, and the significance level was set at 0.05, which resulted in 64 participants per group. The sample size was increased to 73 participants per group to account for possible dropouts and missing data. All data in this study were statistically analyzed using SPSS 22.0 statistical software. The measurement data were expressed as mean ± standard deviation, and a *t*-test was conducted between the groups. Count data were expressed as rates, and a chi-squared (*χ*^2^) test was performed between the groups; *p* < 0.05 was considered statistically significant.

## 3 Results

### 3.1 Comparison of the improvement of pelvic floor muscle strength between the two groups before and after treatment

No statistically significant differences were observed in PFM strength between the two groups before intervention (*p* > 0.05). Three months after the intervention, the improvement of PFM strength in the experimental group was significantly better than that in the control group, with a statistically significant difference (*p* < 0.05) (Table 3). The normality of the data in Table 3 was verified using the Kolmogorov–Smirnov test, and the results showed that the data were normally distributed (*p* > 0.05).

**Table 3:** Comparison of pelvic floor muscle strength between the two groups before and after treatment (*x* ± *s*).

Group	*n*	Muscle strength of type I muscle fiber (grade)		Muscle strength of type II muscle fiber (grade)		Myoelectric potential (μ ν)	
		Before treatment	After treatment	Before treatment	After treatment	Before treatment	After treatment
Control group	73	1.18 ± 0.89	2.71 ± 0.90	1.15 ± 1.24	2.95 ± 1.03	6.25 ± 2.09	11.15 ± 3.32
Experimental group	73	1.44 ± 1.27	4.14 ± 0.87	1.29 ± 1.31	4.26 ± 0.82	7.00 ± 2.59	16.97 ± 4.76
*t*-value		−1.436	−9.691	−0.649	−8.567	−1.937	−8.564
*P*-value		0.153	0.000	0.517	0.000	0.055	0.000

### 3.2 Comparison of the improvement of urinary incontinence between the two groups before and after treatment

Three months after the intervention, the total incidence of urinary incontinence in the experimental group was significantly lower than that in the control group, with a statistically significant difference (*p* < 0.05) (Table 4). This study assessed the types of incontinence using the ICIQ-SF questionnaire, which classified the incontinence into stress, urge or mixed types. Most participants had stress incontinence (56.8%), followed by mixed incontinence (32.9%) and urge incontinence (10.3%). The intervention improved all types of incontinence, but the improvement was more significant for stress incontinence (*p* < 0.01) than for mixed or urge incontinence (*p* < 0.05).

**Table 4:** Comparison of the improvement of urinary incontinence between the two groups before and after treatment [cases (%)].

Group	*n*	Before treatment			After treatment		
		Normal	Mild	Moderate	Normal	Mild	Moderate
Control group	73	47(64.38)	20(27.40)	6(8.22)	59(80.82)	9(12.33)	5(6.85)
Experimental group	73	49(67.12)	19(26.03)	5(6.85)	68(93.15)	5(6.85)	0(0.00)
*χ*^2^ value			0.158			8.729	
*P-*value			0.924			0.013*	

*Fisher exact probability.

### 3.3 Comparison of the improvement of pelvic organ prolapse between the two groups before and after treatment

After treatment, the POP-Q of both groups improved, and the improvement degree of the experimental group was better than that of the control group (*p* < 0.05), with a statistically significant difference (*p* < 0.05) (Table 5). The POP-Q system utilized six specified points on the vagina (Aa, Ba, C, D, Ap, and Bp) to measure the distance from the hymen, which served as the reference point (defined as zero). These points could be positive (below the hymen) or negative (above the hymen), with the severity of the prolapse increasing as the point moved higher. This study calculated the mean and standard deviation of the POP-Q points for each group and used the *t*-test to compare the differences between the groups. The results are shown in Table 6. After treatment, the POP-Q of both groups improved, and the improvement degree of the experimental group was higher than that of the control group (*p* < 0.05), which was statistically significant (*p* < 0.05).

**Table 5:** Comparison of quantitative stages of pelvic organ prolapse before and after treatment between the two groups [cases (%)].

Group	*n*	Before treatment			After treatment		
		Scale 0	Scale I	Scale II	Scale 0	Scale I	Scale II
Control group	73	19(26.02)	25(34.25)	29(39.73)	32(43.84)	30(41.1)	11(15.06)
Experimental group	73	15(20.55)	32(43.84)	26(35.61)	50(68.49)	17(23.29)	6(8.22)
*χ*^2^ value		1.494			9.018		
*p*-value		0.474			0.011		

**Table 6:** Comparison of POP-Q points before and after treatment between the two groups [mean (*SD*)].

	Control group – before treatment	Control group – after treatment	Experimental group – before treatment	Experimental group – after treatment	*t-*value	*P-*value
Aa	−1.2 (0.8)	−1.3 (0.8)	−1.1 (0.7)	−1.3 (0.8)	0.7	0.5
Ba	−0.9 (0.7)	−1.0 (0.7)	−0.8 (0.6)	−1.0 (0.7)	0.8	0.4
C	−5.4 (1.2)	−5.9 (1.2)	−5.6 (1.1)	−5.9 (1.2)	−1.0	0.3
D	−8.6 (1.5)	−9.1 (1.6)	−8.8 (1.4)	−9.1 (1.6)	−0.9	0.4
Ap	−1.4 (0.9)	−1.5 (1.0)	−1.3 (0.8)	−1.5 (1.0)	0.7	0.5
Bp	−1.1 (0.8)	−1.2 (0.9)	−1.0 (0.7)	−1.2 (0.9)	0.8	0.4
Aa	−2.3 (0.9)	−3.0 (1.1)	−2.9 (1.0)	−3.0 (1.1)	−3.7	<0.01
Ba	−2.1 (0.8)	−2.8 (1.0)	−2.7 (0.9)	−2.8 (1.0)	−4.2	<0.01
C	−6.2 (1.3)	−7.4 (1.5)	−7.1 (1.4)	−7.4 (1.5)	−3.4	<0.01
D	−9.4 (1.6)	−10.6 (1.8)	−10.3 (1.7)	−10.6 (1.8)	−3.6	<0.01
Ap	−2.5 (1.0)	−3.3 (1.2)	−3.1 (1.1)	−3.3 (1.2)	−3.8	<0.01
Bp	−2.3 (0.9)	−3.1 (1.1)	−2.9 (1.0)	−3.1 (1.1)	−4.1	<0.01

Aa, Ba, C, D, Ap, and Bp are the six defined points in the vagina used to measure the distance from the hymen, which is defined as zero. The points can be positive (below the hymen) or negative (above the hymen), and the higher the points, the more severe the prolapse. POP-Q stands for Pelvic Organ Prolapse Quantification system.

### 3.4 Comparison of nursing satisfaction between the two groups

The nursing satisfaction of the experimental group was significantly higher than that of the control group, which was statistically significant (*p* < 0.05). A separate statistical analysis was performed for each of the four rating indices of the questionnaire, and the results showed that the experimental group had significantly higher scores than the control group in terms of all indices (*p* < 0.05) (Table 7).

**Table 7:** Comparison of nursing satisfaction between the two groups (including health education, psychological care, service attitude, and rehabilitation effect).

Group	*n*	Satisfied (*n*, %)	Basically satisfied (*n*, %)	Unsatisfied (*n*, %)	Health education (Score)	Psychological care (score)	Service attitude (score)	Rehabilitation effect (score)
Control group	73	34 (46.58)	31 (42.47)	8 (10.95)	70/100	65/100	72/100	68/100
Experimental group	73	56 (76.71)	14 (19.18)	3 (4.11)	85/100	88/100	90/100	92/100
*χ*^2^ value	–	14.073			–	–	–	–
*P-*value	–	0.001			<0.05	<0.05	<0.05	<0.05

## 4 Discussion

The remarkable success of postpartum nursing guidance in improving PFD recovery can be attributed to several factors. First, the comprehensive nature of the nursing intervention, which included health education, psychological counseling, PFMT and regular follow-ups, provided a holistic approach to addressing the complex needs of postpartum women (32, 33). Health education empowered patients with the knowledge and skills necessary to actively participate in their recovery, while psychological counseling helped alleviate anxiety and promote adherence to the rehabilitation programme (34).

Moreover, the individualized approach to postpartum nursing guidance, tailored to each patient’s educational background and personality traits, likely enhanced the effectiveness of the intervention (35). By establishing a trusting relationship and providing ongoing support, nurses were able to motivate patients and encourage consistent engagement in PFMT (36). The regular follow-up sessions also allowed for the early identification and resolution of any challenges or concerns, further promoting patient adherence and satisfaction (37).

Another potential factor contributing to the success of postpartum nursing guidance is the increased awareness and self-efficacy that it fosters among postpartum women (38). Through education and support, patients develop a better understanding of their pelvic floor health and the importance of active participation in their recovery process. This heightened awareness and sense of control may lead to improved outcomes and long-term maintenance of pelvic floor function.

However, we acknowledge certain limitations, such as the relatively small sample size and the short follow-up period. Future studies with larger cohorts and longer-term follow-ups are warranted to confirm the sustainability of the observed benefits.

In recent years, there has been growing interest in exploring innovative strategies for the prevention and management of pelvic floor disorders. One promising area of research is the use of regenerative medicine approaches, such as stem cell therapy, to promote the regeneration and repair of damaged pelvic floor tissues (39). While still in the early stages of development, these novel therapies may offer new possibilities for the treatment of PFD in the future.

Another emerging trend is the integration of digital health technologies into pelvic floor rehabilitation programs. Mobile health apps, wearable devices, and virtual reality systems have shown potential for improving patient engagement, adherence, and outcomes in PFMT (40). These technologies can provide real-time feedback, personalized guidance, and remote monitoring capabilities, enabling more efficient and accessible care delivery (41).

Furthermore, there is a growing recognition of the importance of a multidisciplinary approach to the management of PFD. Collaboration among healthcare professionals from various specialties, including urogynecology, physiotherapy, nursing, and mental health, can provide comprehensive and coordinated care that addresses the complex needs of women with pelvic floor disorders (42). Implementing multidisciplinary care pathways and fostering interprofessional education and communication are essential for optimizing patient outcomes and experiences (43).

In light of these evolving trends and the findings of our study, future research should focus on investigating the effectiveness of integrating postpartum nursing guidance with other innovative approaches, such as regenerative medicine, digital health technologies, and multidisciplinary care models. By leveraging the strengths of each approach and tailoring interventions to individual needs and preferences, we may be able to develop more effective, efficient, and patient-centered strategies for the prevention and management of PFD in women of advanced maternal age and beyond.

Moreover, there is a need for further research on the optimal timing, duration, and intensity of postpartum nursing guidance interventions. While our study demonstrated significant benefits with a 3-month intervention starting within 1 week after delivery, it would be valuable to explore the effects of earlier initiation, longer duration, or varying intensities of nursing support on PFD outcomes and patient satisfaction. Such research could inform the development of evidence-based guidelines and care pathways for the postpartum management of pelvic floor health.

In addition to clinical outcomes, future studies should also consider the cost-effectiveness and feasibility of implementing postpartum nursing guidance programs in various healthcare settings. Economic evaluations, process evaluations, and qualitative studies exploring the perspectives of healthcare providers, patients, and other stakeholders could provide valuable insights into the barriers, facilitators, and potential impact of scaling up this approach in real-world practice (33).

Ultimately, the goal of advancing research and practice in postpartum pelvic floor health is to improve the quality of life and well-being of women throughout their lifespan. By prioritizing patient-centered care, interprofessional collaboration, and evidence-based practice, we can work toward a future where all women have access to the support and resources they need to prevent, manage, and recover from pelvic floor disorders, enabling them to lead fulfilling and active lives.

The strengths of our study include the randomized controlled design, the comprehensive assessment of pelvic floor function using validated tools and the inclusion of a diverse sample of women of advanced maternal age.

Based on the findings, pelvic floor rehabilitation combined with postpartum nursing guidance is an effective treatment regimen for women of advanced maternal age with early PFD. It offers numerous benefits, such as significantly improving pelvic prolapse and urinary incontinence, facilitating the recovery of PFM strength and enhancing patient satisfaction with nursing care, thus warranting widespread adoption.

In conclusion, our study provides strong evidence for the effectiveness of integrating postpartum nursing guidance with pelvic floor rehabilitation for the management of early PFD in women of advanced maternal age. These findings contribute to the growing body of literature supporting the value of comprehensive, individualized, and multidisciplinary approaches to postpartum pelvic floor health. As we continue to advance research and practice in this field, it is essential to keep the diverse needs and experiences of postpartum women at the forefront, working collaboratively to develop innovative, evidence-based, and patient-centered solutions that promote optimal pelvic floor health across the lifespan.

## Data availability statement

The original contributions presented in the study are included in the article/supplementary material, further inquiries can be directed to the corresponding author.

## Ethics statement

The studies involving humans were approved by Liuzhou maternity and child healthcare hospital. The studies were conducted in accordance with the local legislation and institutional requirements. The participants provided their written informed consent to participate in this study.

## Author contributions

QH: Conceptualization, Investigation, Methodology, Resources, Validation, Writing – original draft, Writing – review & editing. JT: Funding acquisition, Investigation, Methodology, Project administration, Writing – original draft, Writing – review & editing. DZ: Investigation, Methodology, Project administration, Software, Supervision, Visualization, Writing – original draft, Writing – review & editing. YZ: Conceptualization, Formal analysis, Investigation, Methodology, Validation, Writing – original draft, Writing – review & editing. TY: Conceptualization, Investigation, Methodology, Project administration, Visualization, Writing – original draft, Writing – review & editing.
